# Inflammatory Cytokines and White Blood Cell Counts Response to Environmental Levels of Diesel Exhaust and Ozone Inhalation Exposures

**DOI:** 10.1371/journal.pone.0152458

**Published:** 2016-04-08

**Authors:** Matthew A. Stiegel, Joachim D. Pleil, Jon R. Sobus, Michael C. Madden

**Affiliations:** 1 Duke University Medical Center, Department of Occupational and Environmental Safety, Division of Occupational Hygiene and Safety, Durham, North Carolina, United States of America; 2 United States Environmental Protection Agency, National Exposure Research Lab, Human Exposure and Atmospheric Sciences Division, Research Triangle Park, North Carolina, United States of America; 3 United States Environmental Protection Agency, National Health and Environmental Effects Research Lab, Environmental Public Health Division, Chapel Hill, North Carolina, United States of America; University of Alabama at Birmingham, UNITED STATES

## Abstract

Epidemiological observations of urban inhalation exposures to diesel exhaust (DE) and ozone (O_3_) have shown pre-clinical cardiopulmonary responses in humans. Identifying the key biological mechanisms that initiate these health bioindicators is difficult due to variability in environmental exposure in time and from person to person. Previously, environmentally controlled human exposure chambers have been used to study DE and O_3_ dose-response patterns separately, but investigation of co-exposures has not been performed under controlled conditions. Because a mixture is a more realistic exposure scenario for the general public, in this study we investigate the relationships of urban levels of urban-level DE exposure (300 μg/m^3^), O_3_ (0.3 ppm), DE + O_3_ co-exposure, and innate immune system responses. Fifteen healthy human volunteers were studied for changes in ten inflammatory cytokines (interleukins 1β, 2, 4, 5, 8, 10, 12p70 and 13, IFN-γ, and TNF-α) and counts of three white blood cell types (lymphocytes, monocytes, and neutrophils) following controlled exposures to DE, O_3_, and DE+O_3_. The results show subtle cytokines responses to the diesel-only and ozone-only exposures, and that a more complex (possibly synergistic) relationship exists in the combination of these two exposures with suppression of IL-5, IL-12p70, IFN-γ, and TNF-α that persists up to 22-hours for IFN-γ and TNF-α. The white blood cell differential counts showed significant monocyte and lymphocyte decreases and neutrophil increases following the DE + O_3_ exposure; lymphocytes and neutrophils changes also persist for at least 22-hours. Because human studies must be conducted under strict safety protocols at environmental levels, these effects are subtle and are generally only seen with detailed statistical analysis. This study indicates that the observed associations between environmental exposures and cardiopulmonary effects are possibly mediated by inflammatory response mechanisms.

## Introduction

External environmental stressors are considered to be more closely related to health outcome than any other contributing factor outside of the genome [1,2]. The environment contains a myriad of chemicals that can partition into human biological systems via dermal, inhalation and ingestion routes, and thus influence the health-state by the “environment x genome” interaction [3–5]. The human body also creates and eliminates a variety of metabolic compounds that are found through “discovery” (or non-targeted) analyses of biological media without pre-conception [6–8]. This aggregate chemical space, represented by contaminants from the environment, their metabolites, and endogenous response compounds from life processes within the body, has been defined as the “human exposome” [4].

Barring such broad “discovery” style analyses, however, standard environmental monitoring is generally relegated to a set of “targeted” compounds (usually, those that may be subject to regulation), so the focus of exposure research is narrowed toward specific pollutant-induced biological responses within a person [9–12]. However, human-based intentional exposure studies cannot proceed with the same large ranges of dose that are utilized in other study designs (i.e. *in vivo* non-human animals, microbiome, *in vitro*, or *in silico*) due to safety and ethical concerns [13–15]. In contrast to standard invasive animal and cellular experiments, widely recognized ethical standards permit humans to be purposely exposed to only relatively harmless levels under strict guidelines, often below what might be experienced by the general public in certain industrial or urban areas. As such, the biological or metabolic responses may be correspondingly subtle, especially when the most sensitive populations cannot be studied at higher levels. However, the advantage of using limited deliberate exposures, albeit at low dose-response regimes, is that the exposure is exactly characterized and stable. Therefore, even subtle responses in biologically relevant markers can take statistical meaning.

We note that diesel exhaust (DE) and ozone (O_3_) were chosen for this work as both are subject to ambient air quality regulation; for DE it is via the particulate matter and oxides of nitrogen (NOx; e.g. NO, NO_2_) constituents. We recognize that DE is a complex and potentially variable mixture and so chose to control those exposures using the particulate matter constituent. O_3_ is a secondary pollutant that requires NOx) volatile organic compounds (VOCs) and sunlight to form in the troposphere and so is correlated with DE (during the daytime) as DE has both NOx and VOCs as major constituents [16]. In complex urban airsheds, there are interactions between NO_x_ and O_3_ concentrations; for these exposures, we maintained stable DE and O_3_ concentrations in the face of variable NO_x_. However, the main goal of the study was to examine a “proof of concept” design that would determine whether there were interactions among pollutants in inducing biological responses (e.g., negative, additive, etc). Cross-sectional observational epidemiological studies have shown that DE and O_3_ can create or exacerbate cardiopulmonary health effects in humans, but there is still a need to understand the biological mechanisms that are responsible for any exposure-related biological response [17–23]. *In vivo* non-human animal experiments have more flexibility than their human-based counterparts in that a wider range of exposures are possible. Such studies have shown links between DE-only or O_3_-only exposures, the inflammation caused by these exposures, and other cardiopulmonary responses [24–27]. Stevens et al. 2008, investigated DE exposures on BALB/c mice and found up-regulation of “inflammatory cytokines (IL-1β, CXCL2 [mouse equivalent of IL-8]” and “numerous interleukins and TNF subtypes” in bronchoalveolar lavage fluid (BALF) following the DE exposure [27]. A follow-up study by the same group found that the DE exposure caused an increase in Th2-associated cytokines (IL-5 and IL-10) but not Th1 cytokines (IL-12 and IFN-γ) [28]. This shift, or polarization, toward Th2 (IL-4, IL-5, IL-10, and IL-13) cytokines is indicative of phagocyte-independent allergic immune responses and can also inhibit Th1 cytokine (IL-2, IL-8, IL-12p70, IFN-γ, and TNF-α) synthesis and response (cell-mediated immunity and phagocyte-dependent inflammation) [29–31].

Non-human animal exposure studies have also shown a relationship between changes in the expression of white blood cells (WBCs) following DE and O_3_ exposures [32,33]. WBCs use cytokines to communicate with cells of the immune system, and increases in specific WBCs and cytokines can be linked to inflammatory responses. Kodavanti et al. 2011, studied cardiopulmonary biomarkers of injury in male Wistar Kyoto rats following exposure to DE, O_3_, or combination of DE+O_3_ [32]. Their results showed decreasing concentrations of peripheral lymphocytes following the DE and O_3_ exposures and a significant increase in neutrophils (in BALF) following the DE exposure. The results also showed up-regulation of mRNA expression for some of the injury biomarkers for both the DE and O_3_ exposures. However, the DE+O_3_ co-exposure in this animal study did not display any changes in WBC counts or cardiopulmonary biomarkers of injury possibly indicating some form of complex interactions among the exposures, inflammatory proteins, white blood cells, and mRNA.

There are several human-based controlled chamber studies that address the relationship between DE-only and O_3_-only exposures and the resulting innate immune system response. Holgate et al. 2003, through a series of DE chamber studies, discovered: increased neutrophils and IL-8 mRNA expression (at 300 pg/m^3^ x 1-hour exposure) in and increased lymphocytes (in BALF), IL-8 (in BALF), and neutrophils (in bronchial wash fluid) in a 2-hour exposure at 100 μg/m^3^ [34]. However, they did not see DE exposure-related increases in any of the other measured inflammatory cytokines. Törnqvist et al. 2007, investigated DE exposure (300 μg/m^3^ x 1-hour exposure) on 15 healthy male volunteers and found statistically significant increases in IL-6 and TNF-α 24-hours after the exposure, but no change in circulating neutrophils in plasma [35]. Xu et al. 2013, studied 18 healthy volunteers exposed to 300μg/m^3^ of DE for 3 hours and discovered a significant increase in peripheral blood monocyte and leukocyte counts, but no change in any of the other WBCs or any inflammatory cytokines in blood [36]. Devlin et al. 2012, investigated 2-hour O_3_ exposures at 0.3ppm with 23 healthy volunteers and the results showed statistically significant increases in cytokines IL-1β, IL-8, and TNF-α as well as a statistically significant increase in BALF-measured neutrophils [37]. Alexis et al. 2010, studied 15 healthy volunteers that were exposed to 0.08 ppm of O_3_ for 6.6 hours and found significant increases in sputum-acquired neutrophils, dendritic cells and cytokines (IL-6, IL-8, IL-12p70, and TNF-α) 18 hours after the end of the exposure [38]. Finally, Kim et al. 2011, investigated O_3_ exposure (0.06ppm for 6.6 hours) responses for 59 healthy young adults and saw a statistically significant increase in sputum-acquired polymorphonuclear neutrophils (PMN) following the O_3_ exposures [39].

Given that there is scientific evidence for exposure-related inflammatory responses from separate DE and O_3_ exposures in both humans and rodents, it is prudent to investigate their co-exposures to assess potential synergistic effects [40]. To our knowledge, there are currently no human-based controlled chamber studies that have investigated a mixture of DE and O_3_. In this study, human volunteers and a highly-controlled environmental chamber are used to study exposures to DE, O_3_, and DE+O_3_. Shifts in NO_2_ levels have been noted in various studies of DE as affecting ambient ozone levels, and so we note that the combination of O_3_ and DE may have had some effect on the ultimate outcomes due to modification of NO:NO_2_ ratio in the exposure scenarios [41,42]. This was not part of the study design here, but only be treated as a hypothesis for now.

This study design allows a scripted scenario wherein there is little environmentally related temporal variation where each subject experiences exactly the same exposure levels. Understanding any links between exposures and a biological response is pertinent to understanding the underlying biological exposure-response mechanisms and thus the risk associated with co-exposure to DE and O_3_, especially for the identification of susceptible sub-populations. The overall goal for this study is to explore cells and proteins of the innate immune system (i.e. WBCs and inflammatory cytokines) and their relationship to DE, O_3_, and DE+O_3_ co-exposures. Establishing these links is beneficial in that it will provide evidence in humans of measureable changes in specific blood cell types and provide unambiguous links between exposures and cardiopulmonary effects as mediated by the inflammatory response. Specifically, human subjects were exposed to levels of DE at levels (DE: 300 ug/m3) that could be found in “heavily trafficked drive by” scenarios [43]. O_3_ levels (0.3ppmv) approximated several hours of exposure in heavily polluted cities. These levels are considered relatively safe for short periods of time due to the reversibility of observed biological responses [44,45]. In our case, exposures and co-exposures were two hours long; details are given in the methods section.

Herein, we focus on the effects of the initial exposures and do not investigate longer term “priming” effects for inflammatory response. The second day re-exposures to O_3_ were part of a longitudinal effects study which goes well beyond the initial hypotheses of co-exposures described here. The longitudinal results will be the subject of future articles. Specifically, the present article explores two distinct questions:

1. Do the short-term exposures to DE, O3 or their combination have an effect on the expression of the inflammatory cytokines and white blood cells?
2. Are any exposure-related changes still present 22 hours after the 2-hour long exposure protocol?

The particular suite of cytokines for this study were chosen as they represent a standard multiplex panel used for human inflammatory response assessments available commercially. Certainly, other biomarkers could have been chosen as well; in fact we have used some different markers (including IL-6) in human studies and a suite of markers in cross-mammalian studies for similar convenience of availability [46,47].

## Methods

### Study Population

Briefly, all subjects were recruited into the study under the following criteria: 18–55 years old, healthy (meaning that they did not have a medical condition of any kind), able to exercise for four fifteen-minute increments over a two hour time frame on a recumbent bike at a target minute ventilation rate of 25 L/min estimated body surface area, non-smoking and willing to discontinue use of non-steroidal anti-inflammatory drugs (NSAIDs), vitamin C, and vitamin E.

All volunteer subjects were subjected to standard physical examinations, including blood work and pulmonary function tests, at the on-site EPA clinic in the Human Studies Facility in Chapel Hill, NC, administered by EPA medical staff. The subjects were not food or drink restricted. Table 1 displays the demographics for the study subjects. Participants were informed of the procedures and likely potential risks and each signed a statement of informed consent. Participation was strictly voluntary and subjects could withdraw at any time. The protocol and consent form were approved by the University of North Carolina School of Medicine Committee on the Protection of the Rights of Human Subjects and the US EPA [ClinicalTrials.gov # NCT01874834]. Further description of the protocol can be seen in Madden et al. 2014 (IRB Study #: 09–1344) [48].

**Table 1:** Demographics of study participants for selected samples.

Gender	N	BMI^1^	Age^2^	GSTM1^3^
Male	11	26.5 (24.7–28.3)	27.3 (24.4–30.5)	6+/4-/1nd
Female	4	30.7 (21.7–39.7)	26.2 (22.9–29.9)	3+/1-

^1^ mean (μ) and 95% confidence interval of the μ

^2^ geometric mean (GM) and 95% confidence interval of the GM

^3^ GSTM1 genotype status (+ = positive,— = null, nd = no determination)

### Exposure Scenario: Highly-Controlled Environmental Exposure Chamber

**Fig 1:**
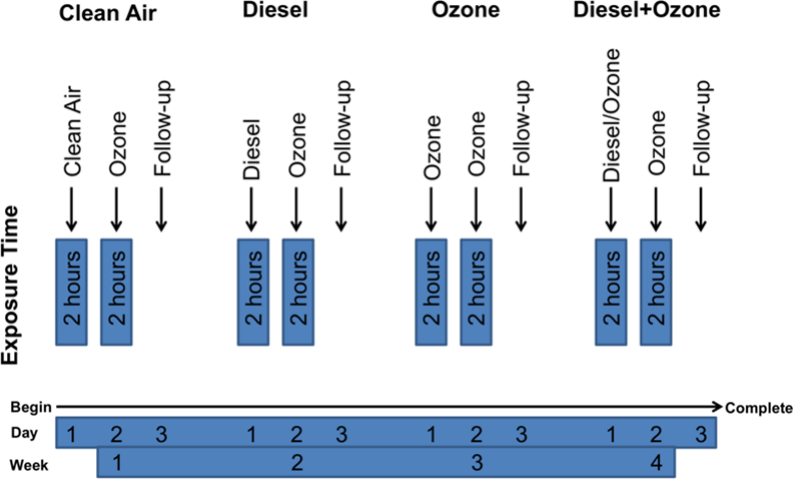
Exposure schedule for volunteers.Each exposure arm is composed of three days. Day 1 is one of the four exposures (clean air, DE, O_3_, or DE+O_3_), Day 2 is always O_3_, and Day 3 has no exposure.

The experimental design for this study was a random-crossover double-blind study with four exposure arms, where each arm was separated by at least thirteen days. Each participant was exposed in an environmentally controlled exposure chamber at the US EPA Human Studies Facility (Chapel Hill, NC) to clean air, DE (300 μg/m^3^), O_3_ (0.3 ppm), and a combination of DE and O_3_ for 2 hours. The environmental exposure order was random, with the participant commencing the study in any of the four exposure arms and progressing though the remaining three arms over the next 2–3 months. The first day of exposure (i.e. clean, DE, O_3_, or DE+O_3_) was always followed by an O_3_ exposure on day 2 and medical follow-up on day 3. **Fig 1**illustrates the four exposure arms and the exposure schedule. As indicated above, this article investigates the relationship between the “Pre” and “Post” samples on Day 1 as well as the “Pre” sample on Day 2, for each exposure; we deliberately restrict discussions to the first 24-hours for each experiment to avoid the potential short term “priming” effects of recent exposures. Pollutant exposure concentrations for PM, O_3_, NOx, SO2, total hydrocarbons (THC), and CO have been previously published and have been included in the S1 Dataset [48].

### Sample Collection and Analysis

180 blood samples were collected from fifteen healthy human volunteers. Whole blood was collected in EDTA-containing 10mL Vacutainer® (Product Number: 368589, Becton, Dickinson and Company, Franklin Lakes, New Jersey) collection tubes, centrifuged, aliquoted, and then frozen at ≤-80°C until analysis. Details of cytokine analyses have been published; briefly, the plasma fraction from the whole blood samples were analyzed using Human Th1/Th2 10-plex Ultra-Sensitive Kits and a Meso Scale Discovery (MSD) SECTOR Imager 2400 (Meso Scale Discovery, Gaithersburg, MD) for the following cytokines: interleukins 1β, 2, 4, 5, 8, 10, 12p70 and 13, IFN-γ, and TNF-α [49,50].

GSTM1 status was determined from isolated peripheral white blood cell DNA using QIAamp DNA real-time polymerase chain reaction Mini Kits (Qiagen Inc., Valencia, CA) and the white blood cell counts were ascertained by LabCorp (Laboratory Corporation of America Holdings, Burlington, NC) [39,51]. All measurement data for cytokines and WBC are provided in the S2 Dataset.

### Statistical Analysis

The 10 cytokines and 3 white blood cells for each exposure and treatment day were tested for normality using Shapiro-Wilks tests (Proc UNIVARIATE, SAS statistical software package version 9.3, SAS institute, Cary, NC). All white blood cells were normally distributed, and cytokine data that were right-skewed were natural log-transformed to satisfy assumptions of normality (see Table 2). The relationships between the pre-exposure (Day 1) and post-exposure (Day 1) cytokine concentrations, and the pre-exposure (Day 1) and follow-up (Day 2) cytokine concentrations for each treatment were investigated using paired student t-tests. Wilcoxin matched-pairs signed rank tests were used for the data that were not normally distributed. P-values <0.05 and 0.05<p<0.10 were considered statistically significant and “moderately” statistically significant, respectively. This analysis had the following *a priori* hypotheses:

1. There will be no change in the expression of the cytokines for the control (i.e. clean) exposure however there will be an increase in the expression of the pro-inflammatory cytokines for the other three (DE, O_3_, and DE+O_3_) exposures.
2. The expression of the pro-inflammatory cytokines in the DE+O_3_ co-exposure will exhibit a synergistic relationship when compared to the single DE or O_3_ exposures.
3. The anti-inflammatory cytokines will show no change in expression for any of the four treatments.

**Table 2:** Descriptive statistics by exposure.^*^

	Clean			Diesel			Ozone			Diesel+Ozone		
Cytokine	Pre	Post	Follow-up	Pre	Post	Follow-up	Pre	Post	Follow-up	Pre	Post	Follow-up
IL-1β	0.440 (0.169,1.15)	0.383 (0.180,0.815)	0.304 (0.158, 0.585)	0.300 (0.139,0.646)	0.347 (0.172,0.703)	0.297 (0146,0.603)	0.499 (0.223,1.11)	0.525 (0.254,1.08)	0.568 (0.264,1.22)	0.442 (0.175,1.12)	0.356 (0.196,0.649)	0.329 (0.152,0.711)
IL-2	0.075 (0.048,0.119)	0.087 (0.057,0.135)	0.082 (0.052, 0.128)	0.094 (0.059,0.149)	0.079 (0.051,0.122)	0.078 (0.048,0.125)	0.127 (0.071,0.226)	0.083 (0.039,0.176)	0.093 (0.054,0.160)	0.078 (0.044,0.139)	0.093 (0.054,0.161)	0.085 (0.055,0.133)
IL-4	0.269 (0.229,0.316)	0.235 (0.206,0.267)	0.271 (0.230, 0.319)	0.312 (0.274,0.364)	0.294 (0.240,0.359)	0.282 (0.241,0.330)	0.450 (0.268,0.758)	0.470 (0.295,0.749)	0.424 (0.254,0.707)	0.321 (0.238,0.434)	0.330 (0.256,0.426)	0.294 (0.213,0.406)
IL-5	0.277 (0.127,0.604)	0.226 (0.115,0.444)	0.245 (0.146,0.413)	0.199 (0.127,0.312)	0.164 (0.114,0.236)	0.196 (0.130,0.296)	0.219 (0.134,0.358)	0.190 (0.116,0.310)	0.253 (0.164,0.392)	0.211 (0.143,0.311)	0.177 (0.125,0.250)	0.231 (0.152,0.350)
IL-8	2.03 (1.66, 2.49)	1.81 (1.52, 2.15)	1.93 (1.61, 2.32)	1.97 (1.61, 2.41)	1.91 (1.56, 2.33)	1.54 (0.910,2.62)	2.31 (1.73, 3.10)	2.40 (1.73, 3.31)	2.04 (1.54, 2.69)	2.11 (1.69, 2.62)	2.05 (1.67, 2.53)	1.97 (1.63, 2.37)
IL-10	1.12 (0.850,1.47)	1.04 (0.800,1.35)	1.07 (0.824,1.40)	1.01 (0.779,1.32)	0.959 (0.721,1.27)	0.977 (0.759,1.26)	1.34 (0.975,1.85)	1.27 (0.940,1.71)	1.18 (0.861,1.61)	1.08 (0.842,1.39)	1.18 (0.758,1.83)	1.03 (0.764,1.38)
IL-12 p70	0.352 (0.275,0.451)	0.379 (0.300,0.479)	0.365 (0.289,0.462)	0.373 (0.292,0.478)	0.359 (0.283,0.456)	0.390 (0.301,0.505)	0.465 (0.322,0.673)	0.417 (0.270,0.644)	0.427 (0.291,0.628)	0.437 (0.329,0.581)	0.369 (0.276,0.494)	0.413 (0.314,0.545)
IL-13	0.723 (0.368,1.42)	0.733 (0.395,1.36)	0.802 (0.419,1.53)	0.797 (0.441,1.44)	0.664 (0.356,1.24)	0.797 (0.447,1.42)	0.838 (0.414,1.70)	0.873 (0.427,1.79)	0.893 (0.462,1.73)	0.906 (0.464,1.77)	0.890 (0.481,1.65)	0.873 (0.451,1.69)
INF-γ	0.252 (0.133,0.478)	0.205 (0.099,0.423)	0.285 (0.128,0.630)	0.272 (0.140,0.527)	0.205 (0.105,0.400)	0.261 (0.135,0.502)	0.201 (0.113,0.356)	0.173 (0.099,0.303)	0.195 (0.116,0.326)	0.317 (0.191,0.526)	0.224 (0.134,0.374)	0.235 (0.150,0.370)
TNF-α	1.84 (1.54, 2.20)	1.79 (1.53, 2.10)	1.93 (1.60, 2.34)	1.94 (1.63, 2.30)	1.70 (1.42, 2.03)	1.70 (1.13, 2.55)	2.16 (1.57, 2.97)	1.94 (1.38, 2.71)	2.07 (1.55, 2.75)	2.10 (1.62, 2.72)	1.73 (1.40, 2.14)	1.98 (1.58, 2.48)

*geometric mean (lower 95% CI, upper 95% CI), pg/mL

The relationships between the pre-exposure (Day 1) and post-exposure (Day 1) white blood cell percentages, and the pre-exposure (Day 1) and follow-up (Day 2) white blood cell percentages in each treatment were investigated using one-way analysis of variance (ANOVA) with Greenhouse-Geisser corrections and Tukey’s post-hoc tests for multiple comparisons. The multiple comparisons used α = 0.05 and multiplicity adjusted p-values [52]. The multiplicity-adjusted p-values are reported for tests that had statistically significant differences. Finally, Spearman correlation coefficients were calculated.

## Results

### Descriptive Statistics for the Four Exposures

**Fig 2:**
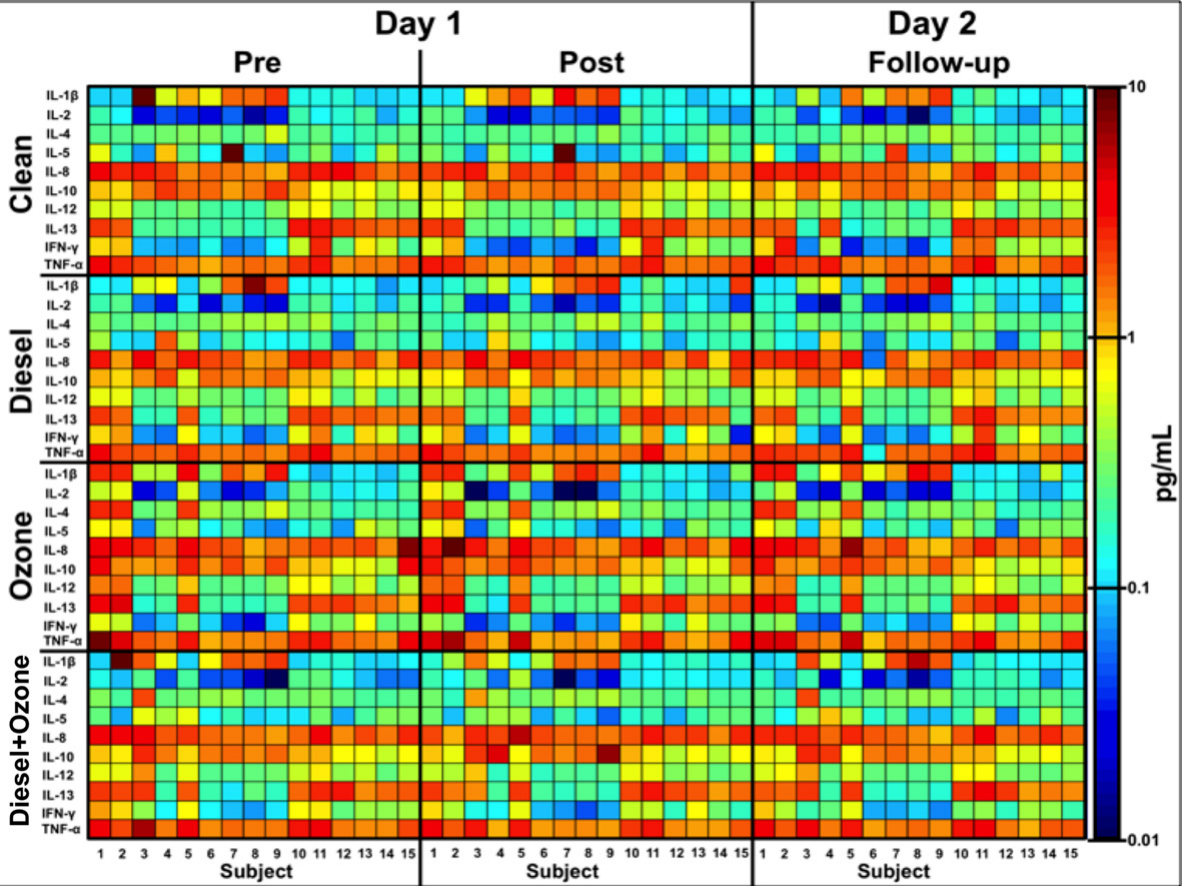
Heatmap of the plasma cytokine levels for all exposure treatments over a 24-hour period. The concentrations in Fig 2 increase as the color changes from blue to red.

**Fig 2**displays the cytokine concentrations for all days and exposures. There are two distinct groups of subjects as well as individual differences. Seven (subjects 3–9) of the 15 subjects have higher concentrations of IL-1β and IL-10 but lower concentrations of IL-2, IL-12p70, IL-13 and IFN- γ when compared to the rest of the group (subjects 1, 2, 10–15). The results for subject 5 indicate a change from the former group (high levels of IL-1β and IL-10) to the latter group for the DE, O_3_ and DE+O_3_ exposures. IL-8, IL-10, and TNF-α have higher sustained concentrations across the two days when compared the other seven cytokines. IL-8 and TNF-α do show decreasing Pre-to-Post concentrations for the DE and DE+O_3_ exposures but not for the O_3_-only exposure where there is a Pre-to-Post increase in concentration. Individual increases for the O_3_-only exposure are also evident. Subjects 2, 3, 10, and 11 have increasing IL-8 and TNF-α concentrations following the O_3_ exposure while subject 15 has decreasing concentrations following exposure.

Descriptive statistics (geometric mean and +/- 95% CI) for each exposure and day are shown in Table 2. The “pre” exposure concentrations for each exposure-arm on Day 1(as seen in Table 1) should be similar to one another, this holds true for all cytokines except for IL-1β on the “Pre”, “Clean” exposure. Looking at Fig 2, these results could indicate an artifact caused by the concentration of IL-1β for subject 3, which is ~10pg/mL while the concentrations are ≤1.0pg/mL for the majority of the other subjects. Subject 3 is also part of the group (Subjects 3–9) that has increased levels of IL-1β and IL-10 through all exposures. Looking across all exposures, IL-8 and TNF- α had the highest mean concentrations while IL-2 had the lowest mean concentration. The majority of the cytokine concentrations in each of the DE, O_3_, and DE+O_3_ treatments decreased following the respective exposure. The 10 cytokines had pre-exposure geometric means (GMs) for the clean, DE, O_3_, and DE+O_3_ exposures of 0.738pg/mL, 0.727pg/mL, 0.862pg/mL, and 0.800pg/mL, respectively, while the “Post” GMs for the clean, DE, O_3_, and DE+O_3_ exposures were 0.669pg/mL, 0.668pg/mL, 0.833pg/mL, and 0.740pg/mL. In each case, there was an overall decreasing pre-to-post trend.

### Exposure Responses–Pre vs. Post vs. Follow-up

As discussed earlier, human studies are relegated to environmental levels as experienced by the general public in the real-world. As such, effects (if any) are subtle and require detailed statistical analysis to be generalizable to larger populations. In the following analyses, we show that despite the large variance components observed, there are indeed statistically significant changes observed in the measured inflammatory markers.

**Fig 3:**
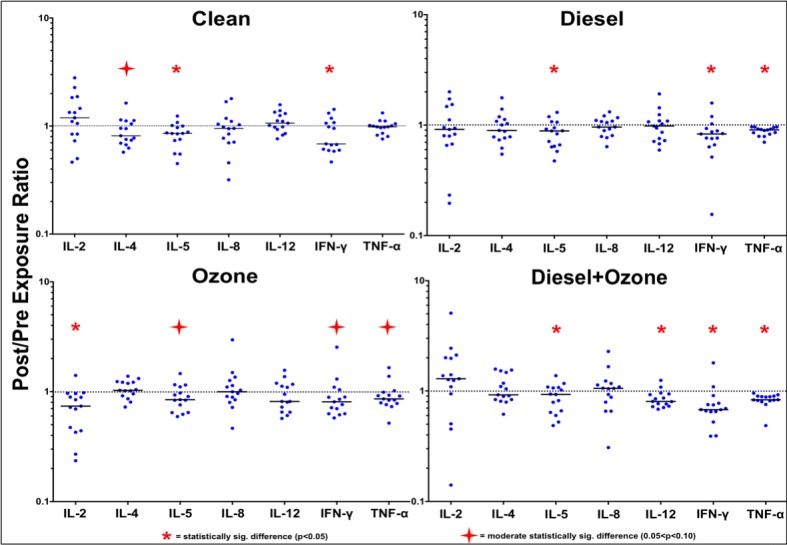
Post/Pre exposure cytokine concentration ratios for the four treatments. Each blue “dot” represents an individual Post/Pre cytokine concentration ratio for that respective treatment. A solid line in each of the cytokine scatterplots displays the median post/pre ratio. The dotted line that is anchored at “1” on each y-axis indicates the ratio where the Pre-exposure concentration is equal to the Post-exposure concentration. A statistically significant result above/below “1” indicates that there has been an increase/decrease in the concentration for that respective cytokine at end of the 2-hour exposure period.

**Fig 4:**
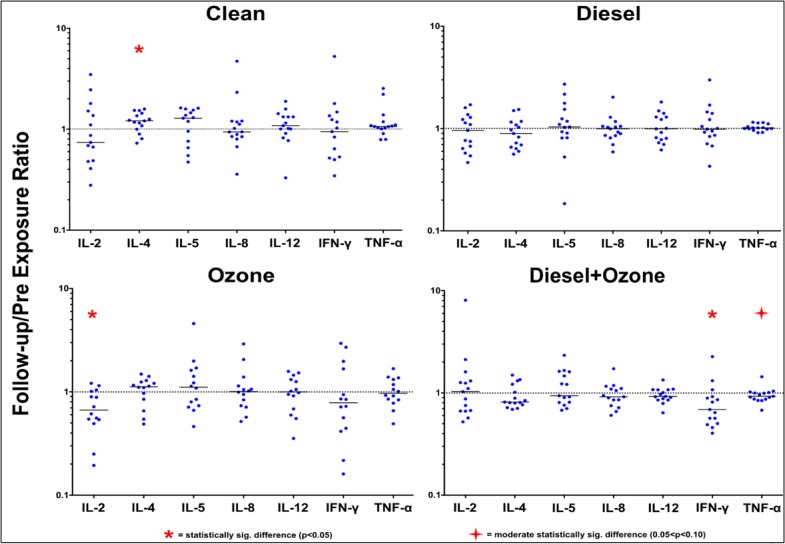
Follow-up/Pre exposure cytokine concentration ratios for the four treatments. Each blue “dot” represents an individual Follow-up/Pre cytokine concentration ratio for that respective treatment. A solid line in each of the cytokine scatterplots displays the median follow-up/pre ratio. The dotted line that is anchored at “1” on each y-axis indicates the ratio where the Pre-exposure concentration is equal to the Follow-up concentration. A statistically significant result above “1” indicates that there has been an increase in the concentration for that respective cytokine 22-hours after the end of the 2-hour exposure period. A statistically significant result below “1” indicates that there has been a decrease in the concentration for that respective cytokine 22-hours after the end of the 2-hour exposure period.

Matched, post-exposure to pre-exposure ratios and follow-up to pre-exposure ratios were created for each exposure and cytokine and displayed in **Figs 3 and 4**. In Fig 3, a ratio above “1” indicates that the “post” exposure concentration was higher than the “pre” exposure concentration and a ratio below “1” indicates the opposite. In Fig 4, a ratio above “1” indicates that the “follow-up” exposure concentration was higher than the “pre” exposure concentration and a ratio below “1” indicates the opposite. Only statistically significant results are reported below; three (IL-1β, IL-10, and IL-13) of the ten cytokines had no statistically significant Pre-to-Post or Pre-to-FU differences.

Given that there was no “exposure” on Day 1 of the clean treatment, minimal Pre-to-Post change was expected following the 2-hour treatment period, but unanticipated results were observed. The two anti-inflammatory cytokines, IL-4 and IL-5, had moderate and statistically significant decreases following the treatment, respectively, and IFN-γ, a pro-inflammatory cytokine, also had a statistically significant decrease. The other pro-inflammatory cytokines (IL-2, IL-8, IL-12p70, and TNF-α) had no statistically significant results. IL-4, IL-5, and IFN-γ all have t-cells as their progenitor cell but IL-2 and TNF-α, the other two cells that can be produced by t-cells, had no statistically significant results. The lack of statistically significant results for IL-5 and IFN-γ in Fig 4 indicates that the cytokine levels return to their “Pre” exposure levels 22-hours after the clean treatment. However, IL-4 switches from a moderate statistically significant Pre-to-Post decrease to a statistically significant 22-hour post treatment increase.

The median ratios for the DE exposure all display a Pre-to-Post decrease. Similar to the clean treatment, IL-5 and IFN-γ have statistically significant decreases in cytokine concentration from the “Pre” values to the “Post” values however TNF-α also has a statistically significant decrease. Unlike the clean treatment, there is no noticeable difference between either the pro- or anti-inflammatory classifications or which cell produces the cytokines of interest. There are no statistically significant results for the follow-up comparison (see Fig 4).

The post-exposure results for the O_3_ exposure show that 5/7 cytokines have Post/Pre ratios that are below “1”, with IL-2 having a significant decrease and IL-5, IFN-γ, and TNF-α having moderately significant decreases (see Fig 3). These results are very similar to the DE results from Day 1; the Post/Pre ratios for the majority of the cytokines decrease however there are few statistically significant results. All of the pro-inflammatory cytokines on Day 1 had decreasing median Post/Pre ratios and the two anti-inflammatory cytokines either had no change (IL-4) or exhibited decreasing (IL-5) median Post/Pre ratios. The Follow-up/Pre (Fig 4) ratios are unremarkable and all cytokines, except IL-2, have 22-hour post exposure concentrations that are similar to the Pre exposure concentrations. Like the Pre-to-Post Day 1 decrease, IL-2 still displays a statistically significant decrease in expression following the exposure; the “post” mean for IL-2 (0.169pg/mL) is not statistically different (p = 0.99) than the follow-up mean (0.143pg/mL). The O_3_ samples also display a greater overall range of individual responses, as evidenced by the increase in scatter across the 7 cytokines.

Day 1 of the DE+O_3_ co-exposure had 5/7 cytokines with decreasing median “Pre” to “Post” concentrations following exposure, with four (IL-5, IL-12p70, IFN-γ, and TNF-α) having statistically significant decreases (see Fig 3). Three (IL-12p70, IFN-γ, and TNF-α) of these four cytokines are pro-inflammatory cytokines. The other two pro-inflammatory cytokines, IL-2 and IL-8, have increasing “Pre” to “Post” concentrations, though neither is statistically significant. Fig 3 also shows that two (IL-12p70 and TNF-α) of the three cytokines that can be produced by macrophage have significantly decreases. As mentioned above, IL-5, an anti-inflammatory cytokine, also has a statistically significant “Pre” to “Post” decrease. The most striking difference between the DE+O_3_ co-exposure and the other three treatments are the Follow-up/Pre ratios seen in Table 3. The follow-up samples for IFN-γ and TNF-α are still significantly and moderately significantly less than the Pre exposure samples. However, IL-5 and IL-12p70, the other two cytokines that have significant pre-to-post decreases no longer display this trend.

**Table 3:** Cell count descriptive statistics by exposure and sample time.^a^

			Exposure			
Day	Sample Time	Cell	Clean	DE	O_3_	DE+O_3_
1	Pre	% Lymph	34.4 (1.53)	36.1 (1.86)	34.7 (1.93)	36.2 (1.67)
	Pre	Abs. Lymph	2.01 (0.140)	1.97 (0.139)	1.87 (0.121)	1.90 (0.143)
	Pre	% Mono	10.1 (0.759)	9.13 (0.593)	10.1 (0.827)	10.3 (0.728)
	Pre	Abs. Mono	0.587 (0.046)	0.507 (0.037)	0.573 (0.067)	0.553 (0.038)
	Pre	% PMN	52.3 (1.69)	51.7 (1.81)	52.2 (1.97)	50.1 (1.68)
	Pre	Abs. PMN	3.18 (0.166)	3.01 (0.158)	3.11 (0.0323)	2.76 (0.146)
	Post	% Lymph	28.7 (2.32)	29.0 (2.35)	30.5 (2.18)	30.0 (2.35)
	Post	Abs. Lymph	1.87 (0.145)	1.80 (0.158)	1.85 (0.126)	1.90 (0.162)
	Post	% Mono	8.73 (0.565)	8.73 (0.539)	9.40 (0.709)	8.47 (0.601)
	Post	Abs. Mono	0.547 (0.038)	0.547 (0.040)	0.580 (0.066)	0.500 (0.037)
	Post	% PMN	60.1 (2.56)	59.7 (2.46)	57.9 (2.23)	59.5 (2.57)
	Post	Abs. PMN	3.85 (0.361)	3.85 (0.364)	3.52 (0.0307)	3.57 (0.288)
2	Follow-up	% Lymph	33.3 (1.82)	34.7 (2.00)	35.6 (1.80)	33.5 (1.51)
	Follow-up	Abs. Lymph	1.83 (0.126)	1.93 (0.147)	1.87 (0.127)	1.80 (0.116)
	Follow-up	% Mono	10.2 (0.903)	9.40 (0.524)	10.5 (0.780)	10.2 (0.698)
	Follow-up	Abs. Mono	0.587 (0.056)	0.560 (0.036)	0.553 (0.047)	0.547 (0.042)
	Follow-up	% PMN	53.1 (1.79)	52.6 (1.90)	50.9 (1.74)	53.1 (1.53)
	Follow-up	Abs. PMN	3.42 (0.240)	3.14 (0.201)	2.69 (0.208)	2.85 (0.147)

^a^mean (+/- standard error), % = percent of total white blood cell count, Abs. = # cells*10^3^/uL, Lymph = lymphocytes, mono = monocytes, PMN = polymorphonuclear neutrophil

Fig 3 also illustrates the overall trend of Th1-mediated cytokine suppression for the DE, O_3_, DE+O_3_ exposures; changes in IL-5, a Th2 produced cytokine and seen across all exposures, are potentially related to the study design (i.e. the incremental exercise during the exposure) and are most likely not indicative of an environmental exposure-related effect. The DE exposure shows IFN-γ and TNF-α having statistically significant decreases following exposure. IL-2 and IFN-γ decrease following the O_3_ exposure, while IL-12p70, IFN-γ, and TNF-α all decrease following the DE+O_3_ exposure. Fig 4 shows that the Th1/Th2 “balance” in the DE exposure returns to baseline 22-hours after exposure, but IL-2 is still suppressed in the O_3_ exposure and IFN-γ and TNF-α are still suppressed in the DE+O_3_ exposure. In summary, there are more exposure-related effects for the Th1 cytokines.

### TNF-α

**Fig 5:**
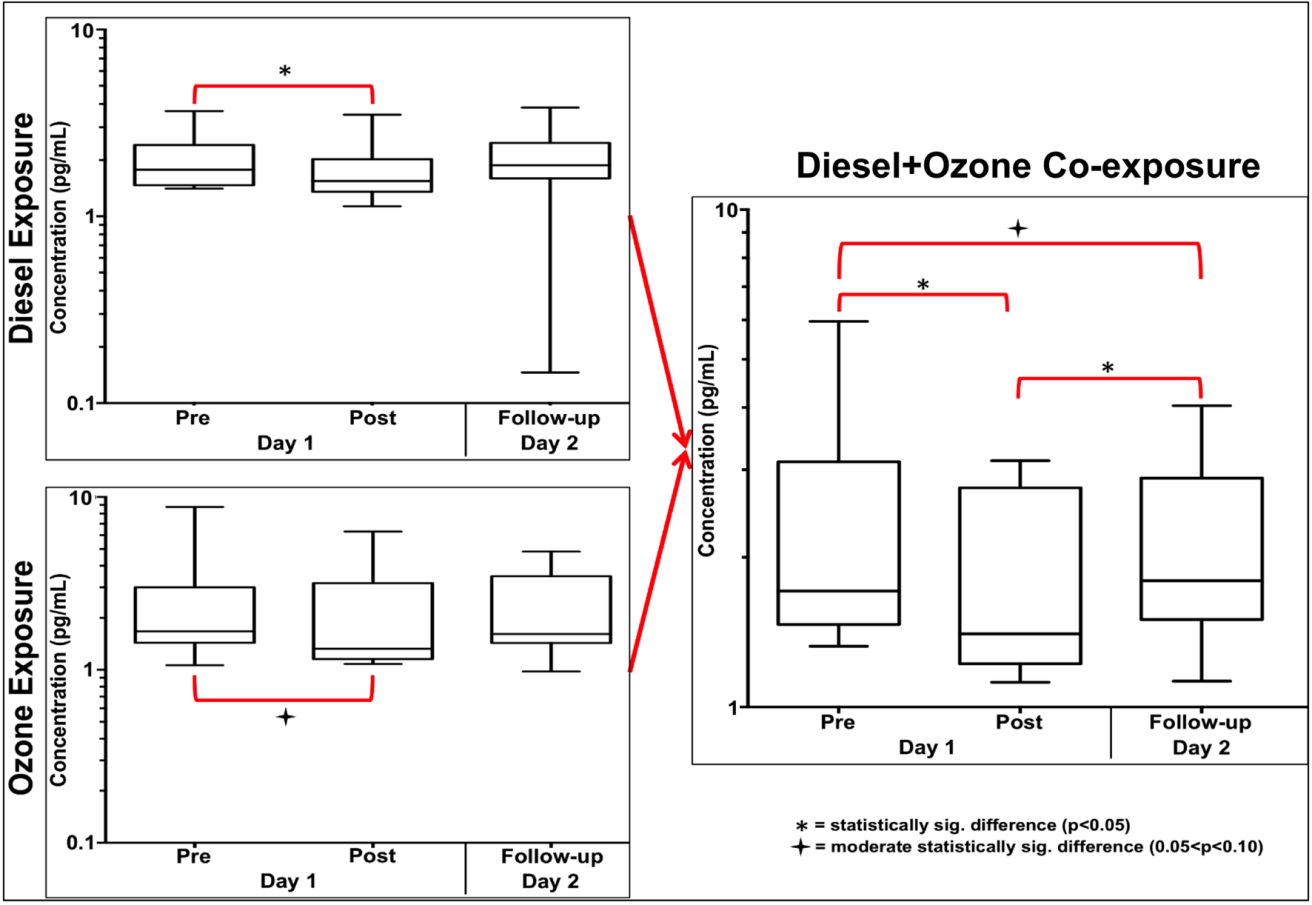
TNF-α cytokine results for three exposure scenarios. The boxes in Fig 5, above, display the median, upper 75%, and lower 25% of the concentrations for TNF-α, while the bars display the upper 97.5% and the lower 2.5% of the concentrations. This figure shows that the combination of the DE-only and O_3_-only exposures into a DE+O_3_ co-exposure creates a suppression of TNF-α that does not recover after a 22-hour period.

The results for TNF-α stand out from the other 6 cytokines for the DE, O_3_, and DE+O_3_ exposures. Fig 5 shows that “Post” concentrations for the three exposures are generally less than the “Pre” concentrations for the respective day. The “Post” concentrations for both the DE and O_3_ exposures are significantly and moderate significantly less than the “Pre” values, for the respective exposures. These two exposures also show that the Follow-up median value is not statistically different from either the “Pre” or “Post” sample from Day 1.

The DE+O_3_ graph on the right side of Fig 5 shows that there are an increased number of statistically significant results when compared to the DE and O_3_ graphs on the left side of the figure. Of particular note, there are no statistically significant results on Day 2 for the DE or O_3_ exposures, but the combination of these two exposures into the DE+O_3_ co-exposure produces a statistically significant result on Day 2. The DE+O_3_ results show that TNF-α is suppressed by the Day 1 exposure and this suppression almost recovers in a 24-hour timeframe. Note that the median for Day 2 is higher than both the Pre and Post median for Day 1, but the moderately significant result still shows an overall pre to follow-up decrease that is similar to the pre to post decrease.

### Cellular Response and Correlations with Cytokines

Table 3 displays the white blood cell count results for the four exposures. The three cell types have “Pre” mean concentrations (as seen in the “Abs. Lymph”, “Abs. Mono” and “Abs. PMN” rows) across the four exposures that are very similar to one another (Table 3). The “Pre” means (# cells 10^3^/uL) and standard error (+/- # cells10^3^/uL) for the lymphocytes, monocytes, and neutrophils for the four exposures are 1.93(0.066), 0.555(0.024), and 3.02(0.106). The percentage calculations display this same “Pre” trend as the absolute (Abs.) values. The “Post” WBC values, both differential and absolute, vary based on the respective exposure. The average absolute lymphocyte counts decreased for the clean, DE, and O_3_ exposures but not for the DE+O_3_ exposure. The DE and O_3_ exposures displayed “Pre” to “Post” increases for the monocytes, while the clean and DE+O_3_ exposures showed average decreases in the absolute monocyte concentrations. All exposures had increasing average PMN concentrations with the DE and DE+O_3_ exposures having the largest increases.

The “Pre” to “Post” differential counts were somewhat different from the absolute concentrations. Changes in the absolute concentrations for some of the WBCs were reflected as a more significant change (and consequently indicated in the statistical tests in Table 4) in the percentage of total WBC calculations. For example, the DE exposure had Pre and Post absolute mean PMN differences of 0.84# cells*10^3^/uL, while the DE+O_3_ exposure had very similar Pre and Post absolute mean PMN differences of 0.81# cells*10^3^/uL. However, this small difference in absolute mean concentration translated into a Pre to Post percentage PMN increase of 8% for the DE exposure and 9.4% for the DE+O_3_ exposure.

**Table 4:** Pre, post, and follow-up statistical comparisons by exposure.^a^

	Exposure							
	Clean		DE		O_3_		DE+O_3_	
	Pre vs. Post	Pre vs. FU	Pre vs. Post	Pre vs. FU	Pre vs. Post	Pre vs. FU	Pre vs. Post	Pre vs. FU
% Lymph	- (0.0025)	none	- (<0.0001)	none	- (0.0306)	none	- (0.00140)	- (0.0270)
Abs. Lymph	none	none	none	none	none	none	none	none
% Mono	- (0.0359)	none	none	none	none	none	- (0.0140)	none
Abs. Mono	none	none	none	none	none	none	none	none
% PMN	+ (0.0010)	none	+ (0.0004)	none	+ (0.0101)	none	+ (0.0003)	+ (0.031)
Abs. PMN	none	none	+ (0.0279)	none	none	none	+ (0.0267)	none

^a^ “+/-” = statistically significant increase/decrease, (p-value), % = percent of total white blood cell count, Abs. = # cells*10^3^/uL, Lymph = lymphocytes, mono = monocytes, PMN = polymorphonuclear neutrophil, FU = follow-up sampling time on Day 2.

Comparing the pre-exposure to post-exposure results shows that the lymphocytes display statistically significant pre-to-post decreases for each of the exposures (see Table 4). The monocytes also have significant pre-to-post decreases but only for the clean and DE+O_3_ exposures. All four exposures have significant pre to post increases for the neutrophil (PMN) differential counts, but only the DE and DE+O_3_ display statistically significant absolute count increases. The DE-only exposure has the largest percentage decrease for the lymphocytes while the DE+O_3_ co-exposure has the largest decrease in percentage for the monocytes and the largest percentage increase in neutrophils.

Unlike the DE+O_3_ co-exposure, the clean, DE-only, and O_3_-only exposures have no significant Pre/Follow-up comparisons. The percentage of lymphocytes in the total WBC count for the DE+O_3_ exposure is still significantly less than the “pre” exposure percentage 22 hours after the exposure, and the follow-up PMN percentages are still significantly more than the pre exposure percentages.

**Fig 6:**
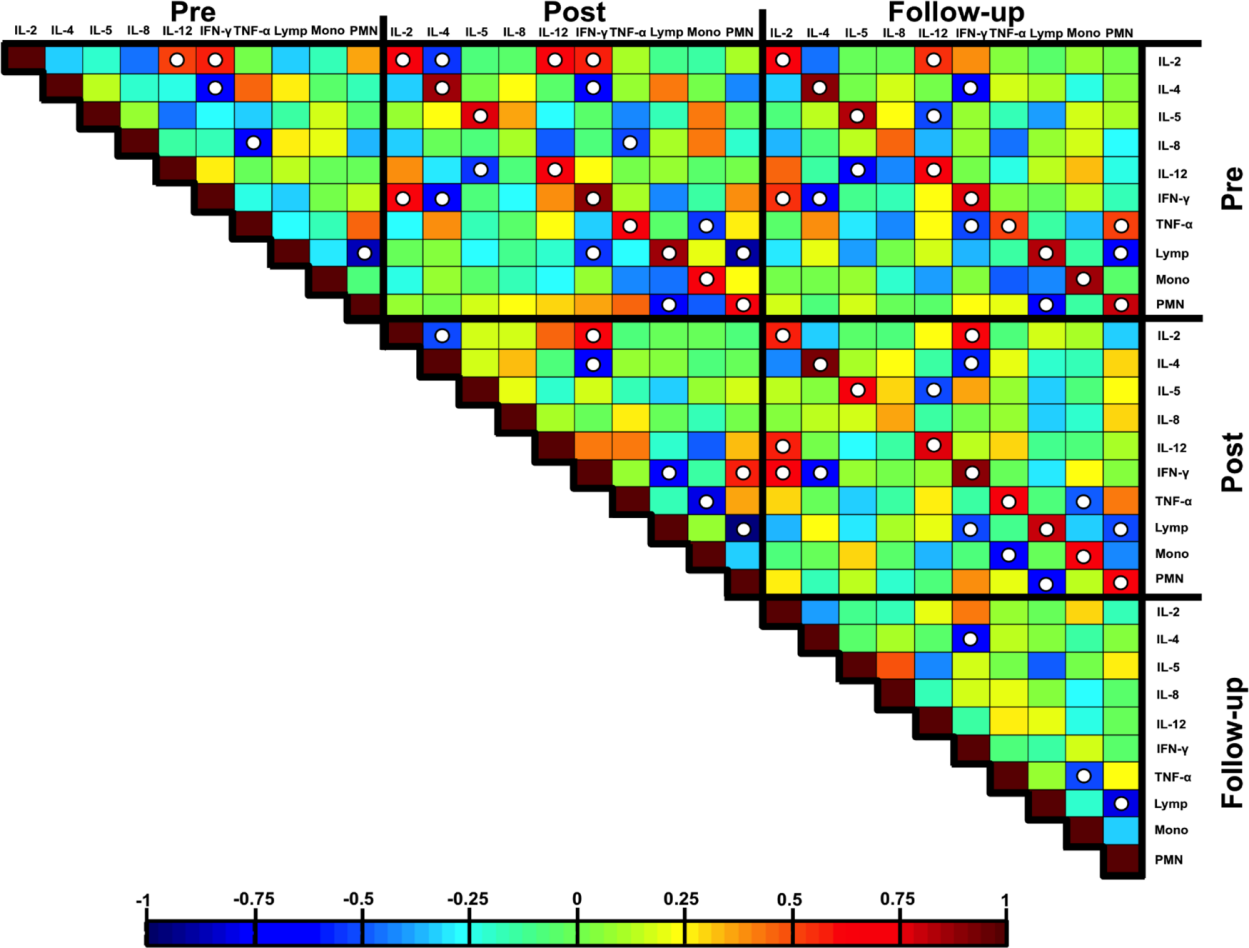
Spearman correlations for pre, post and 22-hour post DE+O_3_ co-exposure responses.A white dot in a cell indicates a statistically significant (p<0.05) positive or negative correlation. Lymph = lymphocytes, Mono = monocytes, PMN = polymorphonuclear neutrophil.

Given the statistically significant results for the DE+O_3_ co-exposure, correlations between the white blood cells and the cytokines were investigated. Fig 6 shows Spearman correlations between the seven cytokines and the three WBCs. Most of the cytokines and WBCs are highly correlated within themselves across the three time period comparisons (i.e. Pre/Post, Pre/Follow-up, and Post/Follow-up). IL-8 is the only cytokine that does not follow this trend. IL-8 does have negative Pre/Pre and Pre/Post correlations with TNF-α, but no other correlations of importance.

IFN-γ has negative correlations with IL-4 for all comparisons, an interesting pro- vs. anti- inflammatory result. IL-12p70 also exhibits some negative correlations but with IL-5, the other anti-inflammatory cytokine. The WBCs maintain the same relationships with one another through all comparisons; the neutrophils are negatively correlated with the lymphocytes and the monocytes have no correlations with either the lymphocytes or the neutrophils. The post-exposure neutrophil percentages are positively correlated with the post-exposure IFN-γ concentrations while the post-exposure lymphocytes are negatively correlated with the post-exposure IFN-γ concentrations. There are no post-exposure relationships for TNF-α with either the lymphocytes or the neutrophils, but it is negatively correlated with the post-exposure monocytes. The correlations between the WBCs and IFN-γ do not persist beyond the post/post comparison, but TNF-α does have a negative correlation with the monocyte percentages for the post/follow-up and follow-up/follow-up comparisons.

Table 3 shows that the mean lymphocytes percentages decrease from Pre to Post. In the same respect, the mean IFN-γ concentrations also display a Pre to Post decrease, however, on an individual basis lymphocyte percentages increase as the IFN-γ concentrations decrease. This is important because the neutrophils are positively correlated with IFN-γ. The relationship is not as strongly correlated (ρ = 0.416 vs. ρ = -0.500 for the IFN-γ/PMN vs. IFN-γ/lymph comparisons, respectively) as the relationship with the lymphocytes, but this example is one of the few described occurrences.

## Discussion

As indicated above, this study had two purposes: one, discover short-term effects on the expression of the inflammatory cytokines and white blood cells, and two, investigate if any exposure-related changes persisted for a day. There was no expectation that the clean exposure would affect the expression of the pro-inflammatory (IL-1β, IL-2, IL-8, IL-12p70, IFN-γ, and TNF-α) cytokines, but a small increase was expected from the DE-only and O_3_-only exposures, with a larger increase expected from the DE+O_3_ co-exposure. There should be little, if any, statistically significant change in the expression of the anti-inflammatory (IL-4, IL-5, IL-10, and IL-13) cytokines for the Day 1 exposures. The expectation for the follow-up results on Day 2 were that any treatment effects from the filtered clean air, DE, or O_3_ on Day 1 would be negligible 22-hours after the exposure. The combination of DE and O_3_ should create the greatest potential to still display a treatment effect; the DE+O_3_ combination could potentially have a synergistic effect as compared to the single DE or O_3_ exposures and create a greater chance having an increase in both pro- and anti- inflammatory cytokines [48,53]. The literature has mixed results for DE exposures, O_3_ exposures, and any effect these exposure might have on changes in lymphocytes, monocytes and neutrophils differential and absolute cell counts. As such, there were no pre-conceived Pre/Post/Follow-up exposure hypotheses for these cells.

We recall that the different arms of the experiments for each subject were conducted separated by multiple weeks so the incoming inflammatory baseline could have been variable depending on intervening events. Furthermore, we also detected perturbations from the “air exposure” arm of the experiments, which we attribute to changes induced from scripted (exercise) activity. To normalize for these variables, we chose to interpret all data “within” experiment and use the “pre” values as a baseline for each exposure.

The heatmap and the descriptive statistics show a heterogeneous effect by the respective treatments for the subjects, as it is evident by the fact that there are two different “groups” of subjects for 6 of the 10 cytokines across all exposures and days. There are also individual results from each exposure, as evidenced by the non-uniform change in the standard error (Table 2) and the range of responses in Figs 3 and 4. These individual changes were expected based on previous research, but the partitioning of the two “groups” was unexpected. The investigation of which personal factors (BMI, age, race, etc.) contribute to this grouping is beyond the scope of this paper, but it is well known that gender can play an important role in the response to environmental contaminants [54]. As such, the differences between male and female responses to the respective exposures were investigated, but the results were inconclusive based on the low number of female participants. All four of the females were in the group with higher concentrations of IL-2, IL-12p70, IL-13 and IFN- γ, (as seen in Fig 2) but there was not enough statistical power to detect a difference between the group of 4 females/4males and the other group of 7 males. In addition, previous research shows genotypic associations (most commonly with GSTM1 +/- status) with proposed differential responses to O_3_-only exposures [51,55]. 4 of the 9 GSTM1- participants were in the group with the higher concentrations of IL-2, IL-12p70, IL-13 and IFN- γ, but similar to the gender investigation, the results were inconclusive. We note that gender and GSTM-1 status may well be important discriminators for O3, but that these data are only suggestive; there are simply not enough subjects in each of these groups for significant statistical analysis.

### Pre, Post and Follow-up Trends

The results from the Day 1 analysis are contrary to the expectations. The statistically significant “Pre” to “Post” decreases for IL-5 and IFN-γ for the clean “exposure” were unanticipated. This change is most likely a bi-product of the incremental exercise on the recumbent bike and/or a circadian cycle variation. This may also be attributable to a “training” effect, but is unlikely as the experimental legs were randomized and a few weeks apart. This potential effect from the exercise is also present on Day 1 of the DE and DE+O_3_ co-exposures, and to some degree the O_3_ exposure, where IL-5 has a moderately significant “Pre” to “Post” decrease. Given past results, it was also expected that the pro-inflammatory cytokines would increase following the DE-only and O_3_-only exposures but the majority of the cytokines had “Post” concentrations that were lower than the “Pre” concentrations. Most of these relationships were not statistically significant, but the decreasing trend for the pro- and anti- inflammatory cytokines was unexpected.

Unlike the results from the other three exposures, the Day 1 DE+O_3_ exposure suppressed both IL-12p70 and TNF-α production. The trend for this type of suppression of an inflammatory response has been seen in DE-only and O_3_-only exposures, but at higher exposure levels and in different biological media [36,56]. The results in this study are the first time that this type of inflammatory response has been seen for a “mixed” exposure and there are a few hypothetical reasons behind this suppression. IL-12 is primarily produced by macrophages and as such, these cells are the most likely candidates for the production of IL-12 given that this study uses an inhalation exposure. Previous research shows that macrophages phagocytize DE particles (DEP), which then influences inflammation [57–59]. In this study, either the DE+O3 combination is limiting the ability of the macrophages to phagocytize the DEP, or the co-exposure is limiting the differentiation of monocytes into macrophages in the epithelial lining of the lung tissue. In either case, the production of IL-12 is limited by the exposure. IL-12 is also known to be precursor and/or stimulating factor for the production TNF-α as well as the recruitment and differentiation of both Th1 and Th2 cells [30]. Thus, the lack of IL-12 expression could play a role in the lack of TNF-α expression as well as some of the other cytokines that have Th cells as their progenitor cell.

With a few exceptions, the Day 2 follow-up results are unremarkable when compared to the Day 1 results as most of the cytokines that were influenced by the Day 1 exposure return to their “pre” exposure levels. IL-4 in the clean treatment does display an increase in expression 22-hours after the exposure, which is the opposite of the trend seen on Day 1, but this can most likely be attributed to the exercise. IL-2 in the O_3_ exposure is still significantly less than the “Pre” sample. This result is one of the few times that this type of suppression has been shown following an O_3_ exposure. The DE+O_3_ co-exposure displays the most striking follow-up results with both IFN-γ and TNF-α still being suppressed. The result for IFN-γ is most likely a combination of the exercise effect, as seen in the clean treatment, and the addition of the suppressive nature on the innate immune system from the DE+O_3_ exposure, which was discussed above for IL-12 and TNF-α.

### TNF-α

The major contrast between the clean “control” and the other three exposures is the change in the expression of TNF-α. This is an expected result as TNF-α is a known pro-inflammatory cytokine and is one of the initial cytokines to be released by macrophages and Th1 cells upon perturbation. However, the statistically significant decreases in TNF-α for the Day 1 DE and DE+O_3_ exposures were unexpected. Fig 5 shows that the DE exposure has a statistically significant decrease (p<0.0001) in TNF-α following the 2-hour DE exposure but did recover to pre-exposure levels by the time the follow-up samples were taken. The DE+O_3_ graph on the right side of Fig 5 shows that the “Pre” to “Post” difference is significant (p<0.0003), and there are two moderately significant results for the Pre-to-Follow-up and Post-to-Follow-up comparisons. Given this, the combination of the DE and the O_3_ seems to have an interaction effect on the exposure and resulting human response, and the direction of the result is an across-the-board significant decrease in TNF-α expression.

As mentioned earlier, a few human-based studies have examined the relationship between TNF-α expression and DE or O_3_ exposure. The results for these studies are mixed with some showing increased expression, a few with decreased expression, and some with no change at all [34–38]. *In vivo* murine studies have also concluded that O_3_ exposure can cause a suppression of TNF-α after an acute dosing regimen. Recently, Oakes et al. 2013, showed that male C57B/L6 mice exposed for 3 hours to an inhalation dose of 2 ppm of O_3_ had a statistical decrease in TNF-α expression when compared to the control [26]. They note that their study, “used a high concentration of O_3_” but that, “exposure to 2 ppm of O_3_ in a rodent was shown to be comparable to 0.2 ppm of O_3_ in a susceptible human subject” [25,26]. The present study has similar exposure metrics (0.3 ppm for 2 hours) and while the DE exposure showed a significant suppression of TNF-α the O_3_ exposure only showed a moderate suppression. Our results for these two exposures are comparable to the current literature, but no studies that have examined relationship between a DE+O_3_ co-exposure and TNF-α expression. Here we have shown for the first time that a DE+O_3_ inhalation co-exposure suppresses the production of TNF-α, and the Day 1 exposure could be a primer for a more involved response on Day 2.

### White Blood Cells and Correlations

The literature has mixed results for the relationship between WBCs and exposures to DE and O_3_, while to our knowledge there are no DE+O_3_ co-exposure results. As seen in Table 3, the “Pre” WBC cell count percentages are equivalent across the four exposures but have varying “Post” changes based on the individual exposure. Changes in the pre-exposure to post-exposure WBC percentages following the clean arm can potentially be attributed to the incremental exercise, a similar result to the relationship between the clean arm and the cytokines IL-5 and IFN-γ. There were also Pre vs. Post statistical differences in the DE, O_3_, and DE+O_3_ exposures, but the magnitude of the differences increased when comparing the DE and DE+O_3_ exposures to the clean treatment. The magnitude of this change, when compared to the clean exposure (the other monocyte decrease), indicates that the combination of the DE and O_3_ are contributory factors. While it could be argued that these relationships are no different than those observed for the clean exposure, the DE+O_3_ Pre-to-Follow-up comparisons show, for the first time, that there is an exposure effect.

Interestingly, the percent changes have less statistically significant Pre/Post and Pre/Follow-up comparisons than the absolute cell count comparisons. These differences demonstrate the shifting immune system dynamics at work related for the specific exposures. For example, the DE+O_3_ exposure has Pre to Post increasing PMN percentages as well as absolute counts, but the absolute lymphocyte counts do not change while the absolute monocyte counts decrease (though not significantly). The percentage decreases for the lymphocytes and monocytes is not due to decreasing overall numbers but the recruitment (and increasing numbers) of PMNs into the peripheral blood following the DE+O_3_ exposure.

Correlations between the WBCs and the cytokines were only investigated for the DE+O_3_ co-exposure given the lack of statistical relationships between the exposure and the cytokine/WBC expression for the other three treatments. The Post/Post comparison between IFN-γ and the lymphocytes displays a strong negative correlation while the neutrophils display a strong positive correlation with IFN-γ. IFN-γ is primarily produced by lymphocytes and the expectation should be that there is a positive correlation between these two, however we see the opposite. This result could be time-related where perfusion from the site of inflammation for the cytokines into the peripheral blood is most likely delayed compared to the increase/decrease of WBC production. However, there were no other time comparisons (i.e. Pre/Post, Post/Follow-up, etc.) that had statistically significant positive or negative correlations between any of the WBCs and IFN-γ.

Finally, the Post/Post and Follow-up/Follow-up anti-correlation between the monocytes and TNF-α is opposite of what would be expected; the peripheral monocyte percentages should be positively correlated with the expression of TNF-α. The results from Tables 2 and 3 show that the mean TNF-α concentrations and monocyte percentages decrease following the DE+O_3_ exposure, but on an individual basis the monocyte counts increase as TNF-α decreases. One conclusion from these results is that there is a time-related explanation similar to that for IFN-γ, however the exposure could also be causing a relative increase in the expression of the monocytes but a relative suppression of TNF-α. While either could be the case, solidifying this association and the relationships between IFN-γ, PMNs, and lymphocytes require further investigation.

## Conclusions

Environmental exposure to air pollutants that could be routinely encountered in urban environments can trigger subtle changes in the inflammatory response in humans. Contrary to expectations, we found that some cytokine messenger proteins are suppressed, especially in response to mixtures of DE and O_3_. We also found that the relationships among cytokines and white cell counts, both related to inflammation, are not uniformly predictable. In short, many of the pre-clinical perturbations in the measured bioindicators are statistically significant, but are difficult to interpret at these environmental exposure levels. From these experiments, we conclude that response/recovery occurs at different time scales making it difficult to identify specific adverse outcome pathways. Furthermore, we found that any pre-clinical effects and perturbations are enhanced with DE and O_3_ co-exposures and so future studies with animal models or with in vitro cell line toxicity tests must take this into account. Finally, there is a subtle exposure “priming” effect on the innate immune system as evidenced by the statistically significant WBC and cytokine changes evident 22-hours after the initial exposures, indicating that future inflammatory response evaluations need to consider previous exposure history.

The question remains as to why there is any interaction effect from the co-exposures at all. Objectively, the O_3_ concentration during the O3-only and DE+O3 exposures remained the same, and the DE particle sizes during the DE-only and DE+O3 exposures were the same at 0.200±0.007μm (Table A in S1 Dataset) [48]. As such, gas exposures and pulmonary deposition should be the same. One remaining measured exposure factor is the co-exposure to NO and NO_2_ from the diesel fuel combustion. The NO:NO_2_ ratio during the DE+O_3_ exposure was 0.03ppm:1.72ppm, which is the inverse of the ratio seen in the DE only exposure (1.58ppm:0.16ppm). This difference in ratios is the one variation seen in the environmental exposure measurements, and so we propose that this shift may be responsible for the increased responses seen in the DE+O_3_ exposure. Additionally there is evidence to suggest that O_3_ interacts with some DE components to alter levels of some volatile organic compounds (VOCs) and semi-VOCs. For 16 VOCs and semi-VOCs (used as surrogates for gas phase organic components) the concentrations monitored during the DE exposure, 4 were greater (styrene, acenaphthene, acenaphthalene, and anthracene), 2 were unchanged (fluorene, naphthalene), and 8 were less compared to the DE+O_3_ concentrations (Fig A in S1 Dataset). These O3+DE changes in the concentrations are approximately 50–100% (increase or decrease compared to DE) with the exception of acenaphthalene (~10X more in the DE). It is unclear that the altered concentrations of these compounds, and likely other gas phase components, in an acute exposure and at these small changes would induce the change in the observed blood responses. Certainly this is purely speculation at this time and this supposition should be tested in more detail in future work.

In summary, the urban exposure scenarios studied herein elicited only subtle perturbations in blood inflammatory signaling, however we caution that the subjects chosen for this work were nominally healthy and between approximately 23 and 37 years of age, thus likely representing a best case scenario in terms of resistance to biological changes. The co-exposures had a possibly synergistic response beyond the sum of the individual exposures, but overall, individual subject heterogeneity made the interpretation of these results difficult. In short, the urban environmental exposures used here showed minimal pre-clinical effects for the outcomes of inflammatory parameters examined and these recovered quickly suggesting that acute effects from such exposures are relatively safe in terms of blood cell and cytokine responses within the healthy general public. This should not be interpreted as a global result with respect to diesel and ozone exposures as we did not consider other potential physiological or preclinical parameters; this was a study only of short-term inflammatory response.

## Supporting Information

S1 Dataset Cytokines and chamber measurement data. (DOCX)

S2 Dataset Exposure chamber measurements for diesel exhaust and O_3_ exposure arms. (XLSX)
