# Supplementary material for: Inflammatory Cytokines and White Blood Cell Counts Response to Environmental Levels of Diesel Exhaust and Ozone Inhalation Exposures
Source: PLoS One. 2016 Apr 8;11(4):e0152458. doi: 10.1371/journal.pone.0152458 (PMC4825980; doi:10.1371/journal.pone.0152458)
Supplement: S1 Dataset — (DOCX) [file pone.0152458.s001.docx]

**S1 Dataset**

**Table A.** Mean day 1 pollutant physicochemical parameters during the O_3_, DE, and DE+O_3_ exposure scenarios^a^.

| Parameter |  |  | O_3_ | DE | DE + O_3_ |
| --- | --- | --- | --- | --- | --- |
| PM (μg/m^3^)^b^ |  |  | 1.0 ± 1.1 | 297. 1 ± 20.8 | 294.3 ± 20.1 |
| PM # (x10^3^/cc) |  |  | <1 | 731 ± 90^b^ (11) | 734 ± 50^c^ (11) |
|  |  |  |  | 977 ± 55^d^ (4) | 903 ± 306^d^ (4) |
| Median PM size |  |  |  |  |  |
| Volume (μm) |  |  | ----- | 0.200 ± 0.007 | 0.200 ± 0.007 |
| Number (μm)^e^ |  |  | ----- | 0.064 ± 0.006 | 0.066 ± 0.003 |
| O_3_ ppm |  |  | 0.30 ± 0.00 | 0.01 ± 0.00 | 0.30 ± 0.00 |
| CO (ppm) |  |  | 0.09 ± 0.10 | 2.58 ± 0.25 | 2.51 ± 0.32 |
| NO (ppm) |  |  | 0.01 ± 0.03 | 1.58 ± 0.26 | 0.03 ± 0.04 |
| NO2 (ppm) |  |  | 0.00 ± 0.00 | 0.16 ± 0.05 | 1.72 ± 0.22 |
| THC (ppm) |  |  | 1.98 ± 0.10 | 2.35 ± 0.14 | 2.24 ± 0.19 |
| SO2 (ppm) |  |  | <0.010 | 0.013 | <0.010 |

mean ± Std Dev except for Median PM size; n = 15, except for PM# where n is indicated.

^a^ Clean air exposure day values were all negligible with the exception of THC value of 2.05 + 0.10 ppm due to the presence of methane (For further description, see Madden et al, 2014; *Particle and Fibre Toxicology* 11:37.).

^b^ Gravimetric weight; average of two teflon filters.

^c^ CPC Model TSI 3022A.

^d^ CPC Model TSI 3775.

^e^ particle size (μm) bin where the median of the particle counts occurred.

**Fig A. Ratio of exposure VOC and semi-VOC components to clean air background^a^.**

**
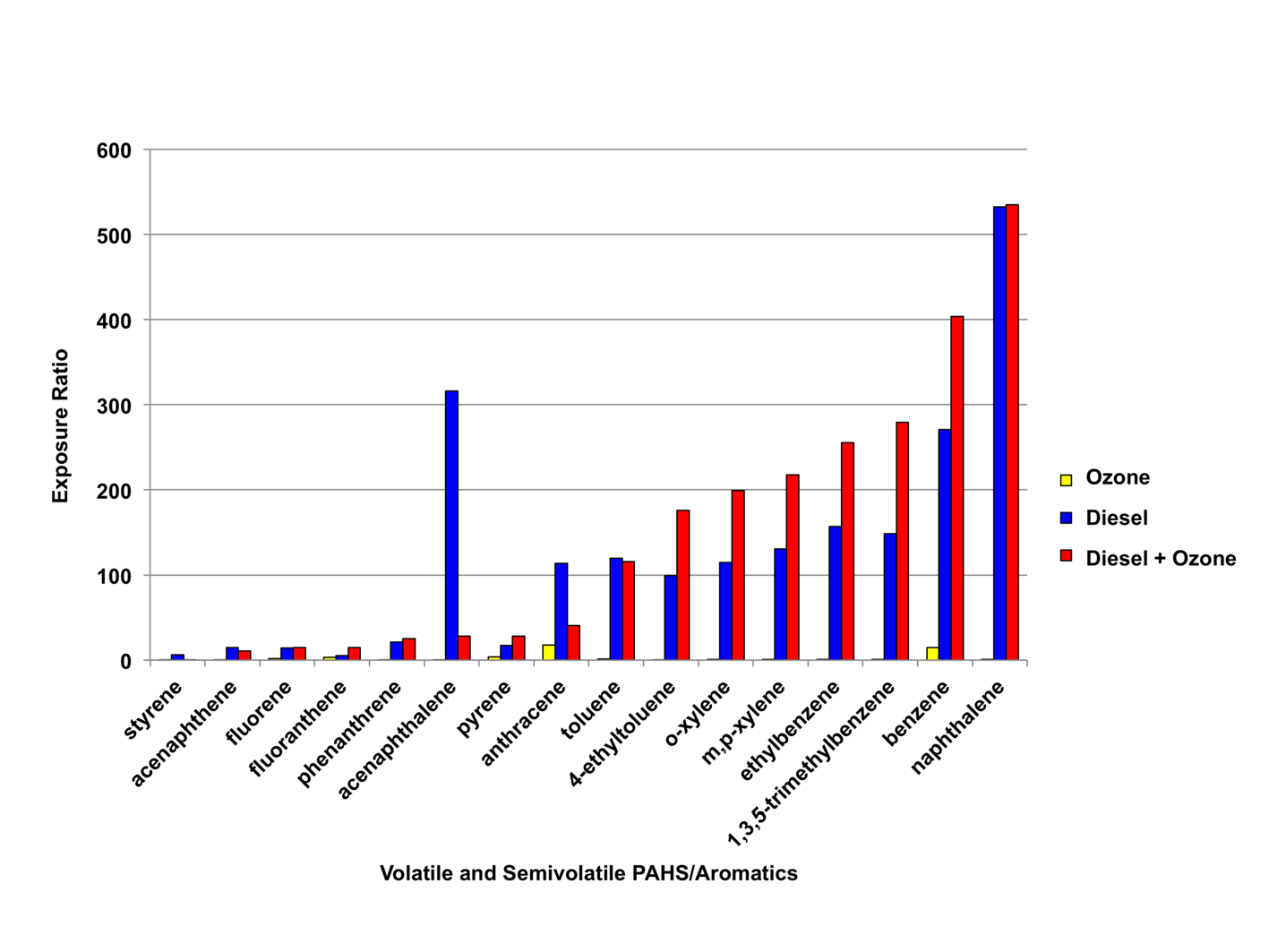
**

**^a^** Samples of chamber air were collected on Tenax cartridges at 100 ml/min during the 2 hr Day 1 exposures. Compounds were eluted from the cartridges at 200^o^C with a helium flow rate of ~2 ml/min. GC-MS Separation and quantitation conditions are provided in Pleil et al. 2014. Component values of the O_3_, DE, and O_3_+DE samples were normalized to the value of the clean air samples [n= 15].

Reference:

Pleil JD, Stiegel MA, and Fent KW. Exploratory Breath Analyses for Assessing Toxic Dermal Exposures of Firefighters During Suppression of Structural Burns. *J Breath Res* 2014; 8(3): 037107.
